# Emergency Field Hospital Surgical Response to Cyclone Chido in Mayotte

**DOI:** 10.1002/wjs.12659

**Published:** 2025-06-19

**Authors:** Anne Daoudal, Jean Philippe Page, Olivier Yavari‐Sartakhti, Guillaume Bouhours, Gauthier Buzancais, Bérangère Sauzeat, Francois Ansart, Jean‐louis Quesada, Paul Ribelles, Yael Lecras, Patrice Dusserre, Valinkini Da Costa, Michel Cherbetian, Isabelle Arnaud, Anthony Couret, Romain Kedzierewicz, Philippe Agopian, Catherine Arvieux

**Affiliations:** ^1^ Department of Vascular Surgery Porte de L'Orient Clinic Lorient France; ^2^ ESCRIM French Civil Protection Field Hospital (EMT‐2) Brignoles France; ^3^ Department of Anaesthesiology Bon Secours Clinic Nancy France; ^4^ Herault Fire and Rescue Department Montpellier France; ^5^ French Civil Protection Unit N°7 Brignoles France; ^6^ Directorate General for Civil Security and Crisis Management Paris France; ^7^ French Military Health Service Paris France; ^8^ Department of Anesthesiology and Intensive Care Angers University Hospital Centre Angers France; ^9^ Health Department Maine et Loire Fire and Rescue Service Angers France; ^10^ Division of Anaesthesia Critical Care Pain and Emergency Medicine Nimes University Hospital University of Montpellier Nîmes France; ^11^ Gard Fire and Rescue Department Medical Sub‐direction Service Nîmes France; ^12^ Department of General and Digestive Surgery Grenoble‐Alpes University Hospital and Grenoble‐Alpes University Grenoble France; ^13^ Pitie‐Salpetriere University Hospital Paris France; ^14^ Gard Fire and Rescue Department Medical Sub‐direction Service Nimes France; ^15^ North Fire and Rescue Department Nîmes France; ^16^ Institute of Advanced Studies of the Ministry of the Interior Paris France; ^17^ Department of Visceral Surgery Regional Hospital of Mayotte Mamoudzou France

**Keywords:** anesthesia, burn, diabetes, global surgery, infection, orthopedics, trauma

## Abstract

**Background:**

The Island of Mayotte was struck by the tropical cyclone CHIDO on 12/14/2024. The French government decided to deploy the Element de Securite Civile Rapide d'Intervention Medicale (ESCRIM) involving a joint detachment of rescue engineers from UIISC7 (Unité d'Instruction et d'Intervention de la Sécurité Civile) and firefighters, comprising a medical support and hospitalization detachment and a surgical support detachment. The aim of this work is to focus on the role of ESCRIM surgical team in Mayotte.

**Methods:**

ESCRIM received 5533 patients between 12/24/2024 and 02/03/2025. A prospective cohort study was conducted from 12/31/2024 to 01/20/2025 on a subset of 130 consecutive patients which underwent surgery in the operating room (OR). Variables included patient demographics, surgical indications, and anaesthetic techniques used. Anonymised data were collected from medical records, surgical reports, and clinical notes.

**Results:**

There were 169 surgical procedures. The mean age of the patients was 31 years, 68% were female, and 27% of procedures were performed in children. General anaesthesia was used in 57% of procedures. The main surgical indications were post‐traumatic extremity septic wounds of the upper limb (38%) and lower limb (27%). 80% of the wounds were grade IV according to the Centers for Disease Control and Prevention Surgical Wound Classification.

**Conclusion:**

This study highlights the importance of adapting surgical practices in resource‐limited settings and the necessity of in‐depth cooperation and coordination among the entire response team and local practitioners. The results of this study contribute to the ongoing challenges of data collection in austere or limited environments.

## Graphical abstract

The Emergency Medical Team ESCRIM (WHO's EMT) was deployed by the French government in the Island of Mayotte hit on 12/14/2024 by the tropical cyclone CHIDO. We conducted a prospective cohort study on 130 consecutive patients, which underwent surgery to describe anesthetic and surgical practices in a situation of massive influx.

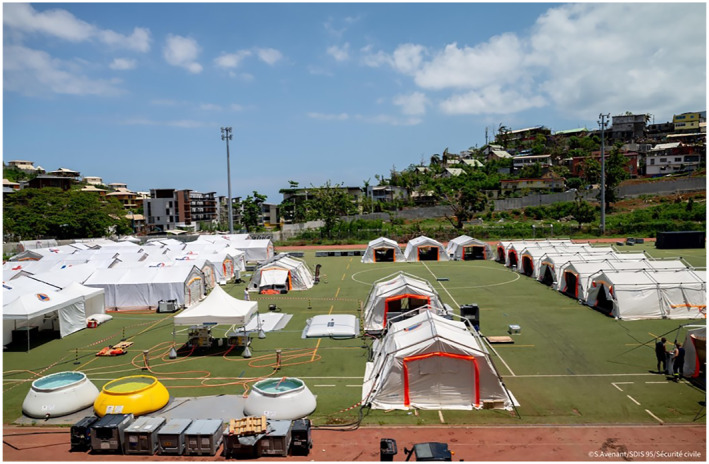

## 1 Introduction

**FIGURE 1:**
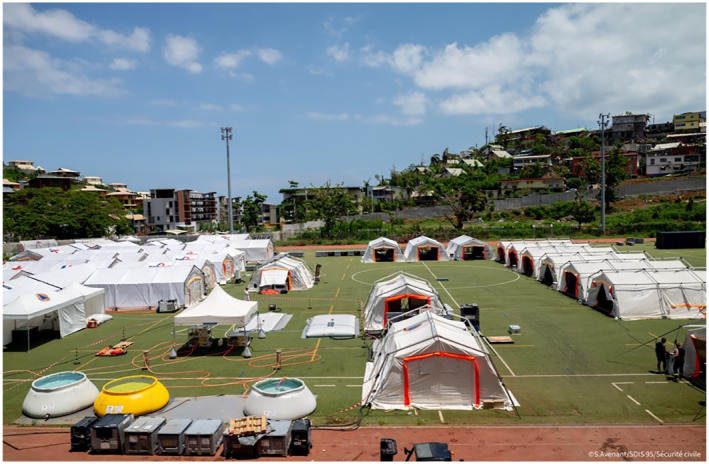
World Health Organization (WHO; Geneva, Switzerland) classified Emergency Medical Team level 2 (EMT 2) Element de Securité Civile Rapide d'Intervention Medicale (ESCRIM) structure deployed in the stadium of Mamoudzou, the economic capital of Mayotte. This module involved a joint detachment of rescue engineers from UIISC7 (Unité d'Instruction et d'Intervention de la Sécurité Civile) and firefighters, comprising a medical support and hospitalization detachment (DAMHO) and a surgical support detachment (DAC). This structure had the theoretical capacity to care for around a hundred people and to perform 7 major or 15 minor operations per day during one month. The DAC consisted of two operating theaters, a sterilization room, four postinterventional monitoring beds (SSPI), six intensive care beds, and two outpatient beds. Due to the partial annihilation of the Mayotte–Dzaoudzi airport, travel from France to Mayotte took respectively 7 days, 3 days et 2 days for the three successive surgical teams to reach the site.

The Island of Mayotte, located in the Comoros archipelago, is densely populated approximately 3000,000‐734 people per km^2^ was struck by the tropical cyclone Chido on 12/14/2024, affecting an already vulnerable population living in informal and precarious settlements known as Bangas [1]. On 12/16/2024, the French government decided to deploy the Element de Securite Civile Rapide d'Intervention Medicale (ESCRIM) to support the emergency services in caring for the victims [2] (Figure 1).

Wild et al. conducted a literature review in 2023 to identify adequacy of records that described perioperative risk assessment in low‐resource or humanitarian settings. The review showed that only 50 reports remained eligible for analysis in quantitative and qualitative synthesis and fewer than half of reports presented the indication for surgery [3]. Similarly, several authors have highlighted the poor quality of reporting and the lack of comprehensive descriptive data on medical activities during natural disasters or conflict‐related humanitarian crises [4, 5, 6].

The aim of this work is to focus on the role of the EMT surgical team in Mayotte in response to the storm and describes their practices in a situation of massive influx.

## 2 Materials and Methods

ESCRIM received 5533 patients between 12/24/2024 and 02/03/2025. We conducted a prospective cohort study from 12/31/2024 to 01/20/2025 on a subset of 130 consecutive patients out of 205, which underwent surgery in the operating room (OR).

Triage of patients followed the Interagency Integrated Triage Tool (IITT) by WHO (https://www.who.int/publications/m/item/IITT) involving the surgeon if necessary. In‐depth discussions with the authorities and the staff of the local hospital of MAMOUDZOU (CHM) showed that their structure, although severely affected by the cyclone, still had a functional maternity ward, CT scan, and two out of five operating theaters, with one dedicated full time for caesarean sections. For patients requiring surgery, the decision was made that only patients with polytrauma and nontraumatic emergencies would be referred to the CHM for CT scan evaluation and treatment. Apart from these few patients, all surgical treatments were carried out at the ESCRIM facility, enabling synergistic re‐evaluation by the surgeon and the anesthetic for the need of reintervention and ongoing antibiotic coverage.

The ESCRIM facility had to be closed from 01/10/2025 to 01/15/2025 due to the occurrence of a second storm. During this period, the population was under curfew with virtually no access to healthcare.

Consent was obtained from all study participants included in the study. Variables included patient demographics, surgical indications, and anesthetic techniques used. Anonymized data were collected from medical records, surgical reports, and clinical notes. Quantitative variables were summarized as means and standard deviations or median {IQR} whereas categorical variables were expressed as number and percentages.

## 3 Results (Table 1)

There were 169 surgical procedures, with 39 patients undergoing two or more procedures over 20 days time period, resulting in a mean of 8.5 surgeries per day (6; 12). The mean age of the patients was 31 years, 68% were female and 27% of procedures were performed in children. General anesthesia was used in 57% of procedures. The main surgical indications were post‐traumatic extremity septic wounds of the upper limb (38%) and lower limb (27%). The majority of the wounds were grade IV according to the Centers for Disease Control and Prevention (CDC) surgical wound classification [7]. Closure by secondary intention was used in 76% of cases, primary closure in 15% of cases, and negative pressure therapy was used in 7% of cases. One transmetatarsal amputation was performed.

**TABLE 1:** Demographic details by procedures.

	*N*
	Mean ± standard deviation (min; max)
	Number (percentage)
Age	31.2 ± 18.3 (2; 74)
Children < 15 years old	46 (27%)
Sex	
Male	115 (68%)
Female	54 (32%)
Localization	
Burns	5 (3%)
Noninfected traumatic wounds	8 (4.7%)
Orthopedic fracture or tendon treatment	2 (1.2%)
Nontraumatic general surgery	8 (4.7%)
Vascular surgery	1 (0.6%)
Lower extremity sepsis	46 (27.2%)
Upper extremity sepsis	65 (38.5%)
Limb root sepsis	20 (11.8%)
Trunk sepsis	14 (8.3%)
Surgical wound classification [7]	
Class I/Clean	6 (3.6%)
Class II/Clean‐contaminated	4 (2.4%)
Class III/Contaminated	23 (13.6%)
Class IV/Dirty‐infected	136 (80.5%)
Anesthesia	
General + intubation^a^	65 (%)
General without intubation with (65 cases) or without intubation (31 cases)	31 (%)
Local	23 (13.6%)
Locoregional	36 (21.3%)
Spinal	3 (1.8%)
Sedation	11 (6.5%)
Types of surgery	
Closure by secondary intention	128 (75.6%)
Direct closure of a wound	25 (14.7%)
Negative pressure therapy on a wound	12 (7.1%)
Other^b^	4 (2.5%)
Number of reinterventions	
0	130 (76.9%)
1	20 (11.8%)
2	9 (5.3%)
3	4 (2.4%)
4	3 (1.8%)
5	1 (0.6%)
6	1 (0.6%)
7	1 (0.6%)
Total of procedures	169

^a^ Major surgery was defined by the WHO as a procedure that requires general anesthesia with intubation and hospitalization [10].

^b^ Four surgeries were performed for nontraumatic indications: 1 case of peritonitis, 1 case of iatrogenic section of the radial artery, 1 case of inguinoscrotal hernia on a 5‐year‐old, and 1 case of infection of a caesarean section.

Perioperative antibiotic treatment, almost exclusively with amoxicillin and clavulanic acid, was carried out for 48 h. Tetanus immunoglobulin injection was administered when the Tetanus Quick Stick test indicated a nonimmunized state.

## 4 Discussion

In terms of daily workload and available equipment, the facility was at the limits of its capacity. Very few surgical patients had to be transferred from ESCRIM to CHM due to the good synergy between the rescue services and the two systems.

Soft tissue and orthopedics procedures were the most frequent, similar to that found in the literature review of Nickerson et al. [8]. We utilized the 2019 Management of Limb Injuries in Disasters and Conflicts consensus to guide clinical care [9]. Decisions about major amputations were delayed to consider patient preference due to the consequences a potentially fatal loss of autonomy and mobility in the living conditions of the Bangas. Interventions were limited to iterative debridement and trimming of necrotic or infected areas.

The main limitations of this study include the lack of long‐term follow‐up and the exclusion of patients who underwent surgery at the structure before December 31, 2024.

## 5 Conclusion

This study highlights the importance of adapting surgical and anesthetic practices in resource‐limited settings and the necessity of in‐depth cooperation and coordination among the entire response team, including emergency physicians, nurses, the operative team, and local practitioners. Despite constraints, it was possible to provide quality care in a complex disaster context. It is essential for teams to recognize that severely infected wounds require specialized care and distinct approaches in the disaster context. The results of this study support following WHO and other expert triage and treatment protocols for disaster contexts and contribute to the ongoing challenges of data collection in austere or limited environments.

## Author Contributions

**Anne Daoudal:** conceptualization, investigation, writing – original draft, methodology, validation, writing – review and editing, supervision. **Jean Philippe Page:** conceptualization, investigation, methodology, validation. **Olivier Yavari‐Sartakhti:** conceptualization, investigation, methodology, validation, writing – original draft, supervision. **Guillaume Bouhours:** investigation, writing – original draft, validation. **Gauthier Buzancais:** investigation, validation. **Bérangére Sauzeat:** investigation, validation. **Francois Ansart:** investigation. **Jean‐louis Quesada:** methodology, data curation, software. **Paul Ribelles:** investigation. **Yael Lecras:** investigation. **Patrice Dusserre:** investigation. **Valinkini Da Costa:** validation, investigation. **Michel Cherbetian:** supervision. **Isabelle Arnaud:** supervision. **Anthony Couret:** supervision. **Romain Kedzierewicz:** supervision. **Philippe Agopian:** supervision, validation, visualization. **Catherine Arvieux:** conceptualization, investigation, writing – original draft, methodology, validation, writing – review and editing, supervision.

## Conflicts of Interest

The authors declare no conflicts of interest.

## Data Availability

The data that support the findings of this study are available from the corresponding author upon reasonable request.
